# Tribological Properties of Chromium Nitride on the Cylinder Liner under the Influence of High Temperature

**DOI:** 10.3390/ma13204497

**Published:** 2020-10-11

**Authors:** Shailesh Kumar Singh, Somnath Chattopadhyaya, Alokesh Pramanik, Sanjeev Kumar, Animesh K. Basak, Shailesh M. Pandey, Qasim Murtaza, Stanislaw Legutko, Grzegorz Litak

**Affiliations:** 1 Department of Mechanical Engineering, Indian Institute of Technology (ISM), Dhanbad, Jharkhand 826004, India; shaileshsonu10@gmail.com; 2 School of Civil and Mechanical Engineering, Curtin University, Bentley 6102, Australia; alokesh.pramanik@curtin.edu.au; 3 Department of Mechanical Engineering, Krishna Engineering College, Ghaziabad 201007, India; sanjeevkg9@gmail.com; 4 Adelaide Microscopy, The University of Adelaide, Adelaide 5005, Australia; animesh.basak@adelaide.edu.au; 5 Department of Mechanical engineering, National Institute of Technology, Patna 800005, India; shaileshmanipandey@dce.ac.in; 6 Department of Mechanical engineering, Delhi Technological University, Delhi 110042, India; qasimmurtaza@gmail.com; 7 Faculty of Mechanical Engineering, Poznan University of Technology, 3 Piotrowo Street, 60-965 Poznan, Poland; stanislaw.legutko@put.poznan.pl; 8 Faculty of Mechanical Engineering, Lublin University of Technology, 20-618 Lublin, Poland; g.litak@pollub.pl

**Keywords:** CrN coating, PVD, reciprocating tribometer, sliding friction, wear, temperature

## Abstract

The chromium nitride coating is a hard coating used to improve the sliding friction and wear behavior and is applied to engine components in various operating conditions even at an elevated temperature. In this study, chromium nitride was deposited by a physical vapor deposition process onto the cast iron substrate. All tribological tests were performed on linear reciprocating tribometer with a stroke length of 5 mm in a dry condition at variable temperature levels of 28 °C, 100 °C, 200 °C, and of 300 °C corresponding to loads of 10 N, 20 N, 30 N, and 40 N against the cylinder liner material. The worn surfaces of chromium nitride(CrN) coatings after friction tests were analyzed by scanning electron microscope (SEM) and energy-dispersive spectroscopy (EDS). The results showed that friction coefficients (COF) ranged from 0.93 to 0.34 from room temperature to 300 °C against the cylinder liner material as a counter-body of 6 mm in diameter; higher temperature results in the positive tribological performance of CrN, with at least 0.34 COF at 300 °C. The wear mechanisms of CrN and counter-body surfaces are abrasive wear accompanied by the slight oxidation. This study guides the wear behavior of cylinder liner coatings in an environment similar to the engine.

## 1. Introduction

Thin-film coating is one of the major spheres of influence among the surafce engineering technologies. There is a critical need for appropriate solutions regarding the friction and wear behavior of the coated alloys. Different types of wear tester are widely used among the researchers mas per the applications of coatings to identify the triboligical behavior. Tribological coatings are frequently characterized with help of the pin-on-disc, block-on-ring, rubber-wheel, micro-abrasion, and abrasion wear tester. It is evident from the literature that reciprocating wear tester provides more accurate results for the piston rings and cylinder liner arrangements in comparison to pin-on-disc wear tester. Piston rings and cylinder liner combinations, along with the crankshaft and piston skirt, are primarily accountable for the frictional losses in the engine, which results in major power loss due to the piston ring-cylinder liner system. Therefore, hard thin-film coatings with superior tribological behavior are required to meet the challenges of engineering applications [1,2,3].

The efficiency of automobile engine components is the attention of the researchers and the liner-ring arrangement plays a vital role by produce mild wear. Surface coating is one of the best methods to control the wear of the materials and many techniques exist to deposit the harder materials such as plasma-assisted chemical vapor deposition (PACVD), physical vapor deposition (PVD), thermal spray processes, diffusion coating processes, etc. PVD is one of the most widely used thin-film coating techniques for intrinsic geometries. It is a deposition technique that includes atomic vaporization followed by deposition of the target material in the form of the coating. A variety of metals, ceramics, and alloys can be deposited by PVD, some of the commonly deposited coatings arechromium nitride (CrN), titanium nitride (TiN), diamond-like carbon (DLC), etc. Titanium nitride (TiN) coating is popular due to itsgalling tendency to forming and cutting tools. Chromium nitride (CrN) is another accepted example of PVD coating that manifest high hardness, high thermal stability, low friction, and high corrosion-resistance properties. CrN is also extensively used in automotive industries, due to its wide applications in lubricated conditions. These is due to the fact of textured surface that provides micro-reservoirs for lubricants and, as a result, reduces the friction and wear [4,5,6,7,8]. The tribology of Al atoms are introduced into TiN to form the AlTiN coating was also investigated at a high-temperature wear tester against a ceramic (Si_3_N_4_) ball with a diameter of 6 mm at load of 5 N, 7 N, and 9 N with a working temperature of 800 °C. The steady-state value of friction coefficient of AlTiN coating ranged from 0.6495 to 0.3898 at above operating conditions. The common wear mechanisms are composed of adhesive, abrasive, and oxidation wear, accompanied by minor fatigue wear [9]. The friction and wear behavior has been reported in lubricated condition for various applications. A trib test has been carried out at 10 Hz and 10 mm stroke length at 100 °C and resulted in abrasive wear as a dominating wear mechanism [10].

Because of the literature as mentioned earlier, most of the wear tests have primarily been investigated at the unidirectionalpin-on-disc tribometer at room temperature. Still, hard thin-film coatings are capable of withstanding higher temperatureand loads as per applications so far, and there is very little in the literature regarding the behavior of such coatings subjected to engine operating temperatures especially for rings and liner pair. Most of the works of literature of internal combustion engine component deal with the pin-on-disc tribometer, while this setup gives appropriate wear and friction identification of rotating engine components. Thus, the proper identification of wear and friction characteristics between the cylinder liner and piston ring pair should use a test setup, i.e., reciprocating type tribometer that is similar to real operational conditions. Therefore, the objective of the present work is to predict the behavior of CrN against cylinder liner in a reciprocating motion at high temperature and dry environment. Thus, results of the study might be very useful to future researchers and professionals to further investigate the applications of this type of coating.

## 2. Methods and Experiments

### 2.1. Materials for Experiment

**Figure 1:**
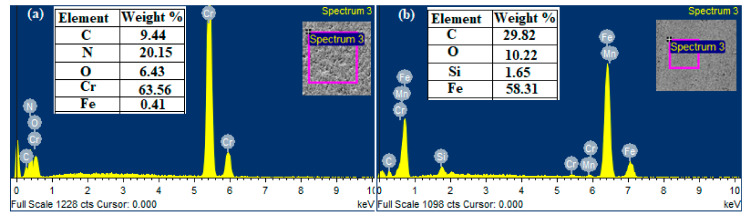
(**a**) The composition of chromium nitride (CrN) sample before sliding wear test by EDS, (**b**) The composition of counter-body before sliding wear test by EDS.

Coating samples (CrN) were fixed in the disc of the sliding wear tester against a counter-body of the cylinder liner material of diameter 6 mm. The composition of the CrN-coated sample has been studied with the FE-SEM equipped with the EDS. It can be clearly seen in the table and spectra the CrN composed of 63.56% chromium (Cr), 20.18% nitrogen (N_2_) by the weight percentage in Figure 1a. While the EDS spectra of the counter-body (pin) represented in Figure 1b before wear test composed of 58.31% iron (Fe) and 29.82% carbon(C) by the weight percentage. The samples were cleaned in acetone with the help of an ultrasonic oscillator and rinsed in ethanol and dried, respectively, before and after wear test for more accurate results of weight loss.

**Figure 2:**
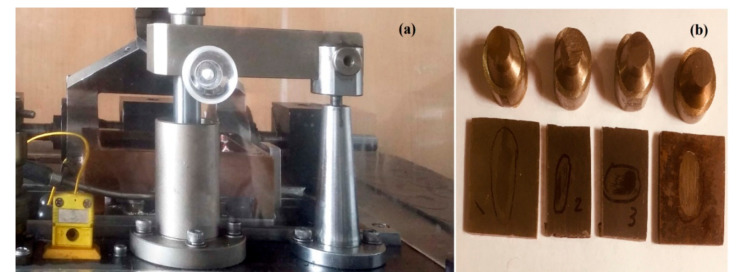
(**a**) Linear reciprocating tribometer chamber (**b**) CrN-coated samples and counter-body after the wear test.

A linear reciprocating tribometer (LRT) having a facility to determine the tribological behavior of the coating has been used. The system moves under linear reciprocating motion with the facility of different normal load (5–50 N), frequency (up to 50 Hz), stroke length (1–20 mm), and temperature (ambient to 550 °C). Wear test has been performed on high-temperature linear reciprocating tribometer at a temperature of 28 °C (room temperature), 100 °C, 200 °C, and 300 °C and the corresponding load of 10 N, 20 N, 30 N, and 40 N (Table 1). The contact pressure kept constant at 5 MPa for the running-in period. It is interesting to see in Table 1 that load and temperature are varying successively. This may lead to reports of the cumulative effect of both the parameters that is missing in the literature. Heater temperature sensor equipped with a j-typethermocouple with least count of 1 °C and accuracy of 1 ± 1% of the measured value in °C. Frequencies, stroke length, speed, and sliding distance remains constant for 1620 s. The condition of the test varies according to the application of piston rings behavior against cylinder liner and partially also according to the literature. The pin of cylinder liner material of 6 mm in diameter was used against the CrN coating. Figure 2a represents the linear reciprocating tribometer chamber to hold the workpiece, and Figure 2b shows the CrN-coated samples and counter-body after wear test. Some stroke difference can be observed inFigure 2b. It is clear from the different tribological studies that sliding of the counter-body against the coating takes a larger force to maintain the sliding that results in the expansion in the junctions or point of contact due to the tangential stress of the surface across the point of contact are the possible reasons of difference in stroke. The evenness of the coated sample and circularity of the counter-body as a pin is a crucial factor during the wear test. The friction coefficient is also unnatural by the normal loads, which in turn affect the various arrangements of sliding wear tester.

### 2.2. Coating Deposition

Ion plating, a physical vapor deposition (PVD) technique was used for periodic bombardment to the substrate results in the deposition of CrN film by atomic-sized energetic particles. This deposition is used for very good adhesion and high-density deposition, modifying coating stress, chemical reactions, and modifying the structure and properties of the deposited thin film. This technique was used to deposit the chromium nitride (CrN) at a temperature of 360 °C. A micro-blasting technique used before coating, gray cast iron as a substrate was cleaned and roughened by using alumina (20–45 µm) particles to eradicate surface irregularities such as dirt, oils, and oxides, etc. to ensure the good bonding of the CrN coating with the substrate. Initially, a Cr layer was deposited to improve the coating adhesion, a furtherCrN layer was deposited with nitrogen (N_2_) as a carrier gas and acetylene under 550 MPa pressure with argon as a fuel gas. The power of the cathode was set at 2600–2800 W, and pulse frequency was adjusted to form nitride phase ofchromium at 1536 °C. The thickness of the CrN layers was controlled by adjusting the deposition time.

### 2.3. Characterization and Analysis Instruments

The wear behavior of CrN coating against the cylinder liner has been analyzed with the help of linear reciprocating tribometer of DUCOM (Bangalore, India) material characterization equipped with winducom 2010 with a maximum temperature limit of 550 °C. The FISCHERSCOPE HM2000 (Pune, Maharashtra) was used to examine the hardness of the CrN-coated sample. The surface and cross-section morphology combined with EDS analysis was measured using a Carl-Zeiss make field-emission scanning electron microscopy (FE-SEM, model—Carl Zeiss Ultra Plus, Roorkee, India). The surface roughness of the coating samples was measured by a scanning probe microscope (SPM) with an atomic force microscope (AFM) make INTEGRA and model NT-MDT-INTEGRA (Roorkee, India). The mass loss of CrN coating and against the cylinder liner as a pin sample was measured using an electronic balance with a least count of 0.00001 g.

## 3. Result and Discussion

### 3.1. Coating Characterization

**Figure 3:**
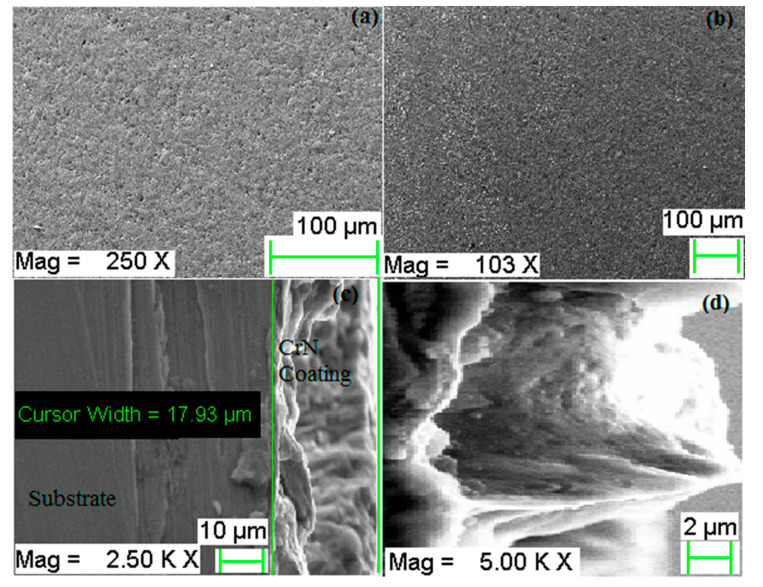
Morphology of CrN coating as deposited, (**a**,**b**) top view of coating at different magnifications, (**c**) cross-section view of coating at 2500×, (**d**) magnified view of a coating thickness area.

The morphology of the coating, as well as the cross-section view of the coating, has been illustrated in Figure 3. The coating consists of well deposited small grains composed of 63.56% chromium (Cr) and 20.18% nitrogen (N_2_) by weight percentage. The coating surface consists of small grains and forms dense structure (Figure 3a) throughout the film with short and disordered columnar texture shown in Figure 3b. The coating thickness of CrN layers is about 17.93 μm (Figure 3c) and the higher magnification view of the cross-section shown in Figure 3d. Neither pin-holes nor voids were observed throughout the structures of the coating.

**Figure 4:**
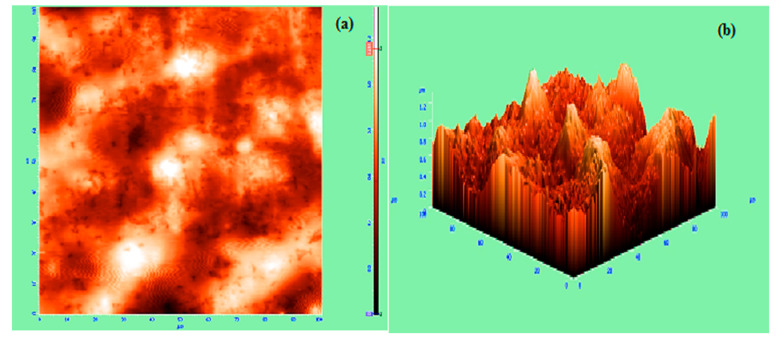
Atomic force microscope (AFM) surface morphologies of CrN film as deposited: (**a**) top view of surface roughness in 2D, (**b**) 3D view of CrN film.

**Figure 5:**
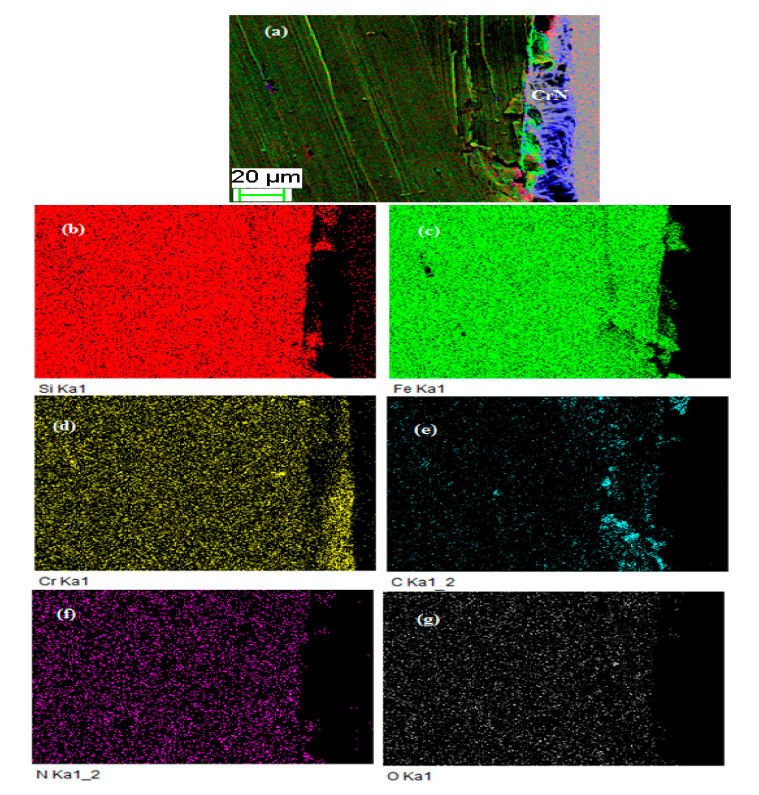
Cross-section mapping results of CrN coating as deposited: (**a**) cross-section scanned position, (**b**) Si content, (**c**) Fe content, (**d**) Cr Content, (**e**) C content, (**f**) N content, (**g**) O content.

The FISCHERSCOPE HM2000 is an automated nano-indentation measuring system has been used to perform indentation test method according to ASTM standard E2546.The hardness of the upper layer of the CrN coating was 681.58 HV, examined at 300 mN/20 s.The detailed structure and phase analysis has been examined with the help of X-ray diffractometry (XRD) of the CrN coatings, which was carried out as reported in our previous corresponds at [11] and confirmed that the structure of the coating is mainly face-centered cubic (FCC) CrN with a trace of hexagonal CrN along (200) and (111) preferential orientation. The average-based parameters were obtained to examine the surface roughness of the coating in Figure 4. It shows the 3D view of coating deposition by atomic force microscope (AFM) and confirms the average roughness (*Sa*) of 142.38 nm with a root mean square (*Sq*) of 178.64 nm. The quoted value of roughness was analyzed for the cut off value from 0.08 to 6 mm. Figure 5 shows the cross-section mapping results of CrN coating as deposited. All the major elements are visible in the mapping, but nitrogen is not traced in the coating region due to its lightness.

### 3.2. Specific Wear Rate

The evolution of wear coefficient was obtained by Equation (1) [12] shown below and denoted by k_o_

k_o_ = W/ρLD (1)

Equation (1) is used to calculate specific wear rate, where ρ is the density of the sample, W is the weight loss, L is the applied load, and D is the sliding distance.

The specific wear rate has been reported in the various literatures at different levels of temperature and concluded that the wear rate was quite higher at 300 °C than that of 500 °C. However some of the coatings showed the stability at higher temperature. The wear coefficient of CrN/QSn7−0.2 was low and reaches to the highest value of 3.83 × 10^−6^ mm^3^/N·m at room temperature because of the strength of the coating to cooperate at a higher temperature [13,14].

The wear rate of coatings depends on many factors like the applied force at point of contact through lever and geometry of the counter-body, sliding velocity, and lubricityat the point/line of the contact. Comparatively at lower speeds and loads, the governing wear pattern is smoothing the top layer of the coating and form lubricious layer of the oxides due to the higher temperature in of wear test chamber.

**Figure 6:**
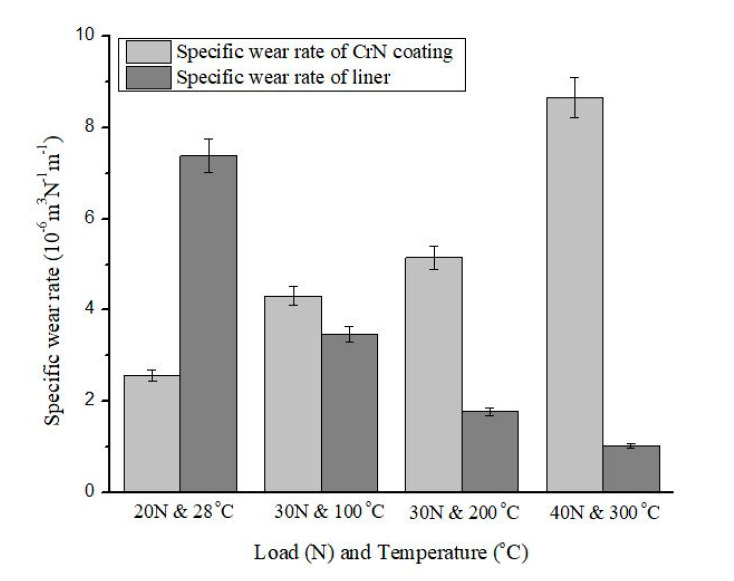
Specific wear rate of coated sample and counter-body at different load and temperature.

The specific wear rate of the coating and counter-body has been represented in Figure 6. The wear coefficient of the CrN coating and counter-body (cylinder-liner) were obtained 2.56 × 10^−6^ m^3^N^−1^m^−1^ and 7.38 × 10^−6^ m^3^N^−1^m^−1^ at room temperature (25 °C), the load of 10 N, and constant sliding speed 0.3 m/s. When a rise in temperature takes place for the remaining test from 100 °C, 200 °C, and 300 °C, the wear coefficient of coating ranges from 4.31 × 10^−6^ m^3^N^−1^m^−1^, 5.15 × 10^−6^ m^3^N^−1^m^−1^, and 8.66 × 10^−6^ m^3^N^−1^m^−1^, whereas for counter-body, it ranges from 3.46 × 10^−6^ m^3^N^−1^m^−1^, 1.77 × 10^−6^ m^3^N^−1^m^−1^, and 1.03 × 10^−6^ m^3^N^−1^m^−1^. Specific wear rate is a function of applied normal load and chamber temperature. It can be seen from the trend of specific wear rate of CrN coating is increasing, which means the coating is not able to retain itself in mild wear condition. This increasing trend of CrN coating can also be supported by the SEM image of the wear track at 40 N and 300 °C. The wear track at this level shows the maximum damage of the coating.

The highest wear may be due to the adhesive junctions between asperities at some stage in sliding wear test. The irregular features on the SEM image of wear track also confirmed adhesive wear. Due to the sliding action, some transient layer may be removed, but some new formation of asperities growth takes place at higher temperature and also facilitates the sticking or formations of some atomic bonds. The higher wear properties of the CrN coating is strongly reliant on the structure of the coating and is mainly face-centered cubic (FCC) with a trace of hexagonal CrN along (200) and (111) preferential orientation. As Figure 6 depicts, that the cumulative effect of the load and temperature confirmed an increase in the wear rate, this is due to fact of instability of the FCC structure of CrN coating at higher temperature. The combined effect of normal load and the temperature of the closed chamber results an increase in wear rate of coating, while decreases in wear rate of cylinder liner material as a counter-body.

Whereas the specific wear rate of the liner as a counter-body showed the highest wear rate at room temperature. Wear of liner characterized by highest wear rate at room temperature with important build-up rates. Interestingly, the wear rate of the counter-body decreased by a rise in temperature. However, the significant change has been observed in wear rate of the pin by an increase in temperature from 28 °C to 100 °C. This may be due to the absence of build-up, which can be considered as a temperature activation process. Further, it is showing a considerable decrease in wear rate of the pin at 200 °C and 300 °C.

### 3.3. Evolution of Coefficient of Friction at Interface

The coefficient of friction CrN coating (5 µm thickness) decreased from 0.80 to 0.57 by an increase in temperature from room temperature to 500 °C against the counter-body of Si_3_N_4_ as ball. While some of the literatures also reported the value of COF of CrN coating at 25 °C and 200 °C are 0.8 and 0.6. On the other hand, it dropped to 0.4 at 315 °C, and then progressively, it was reduced to the minimum value of 0.3 at the highest temperature of 500 °C [15,16].

**Figure 7:**
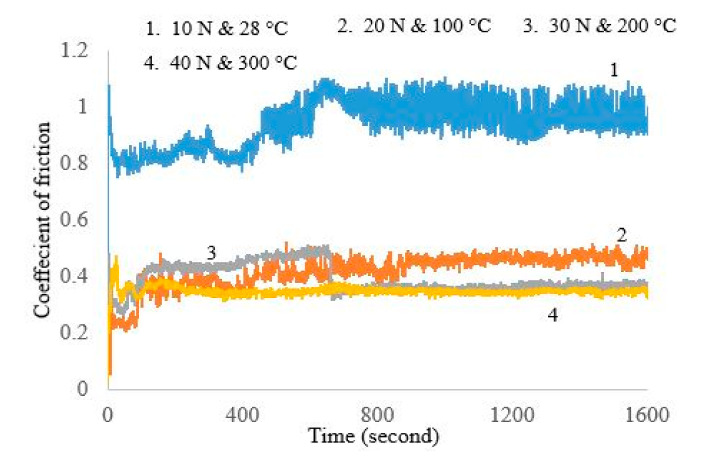
Friction coefficients (COF) at a constant speed with increasing load and temperature.

**Figure 8:**
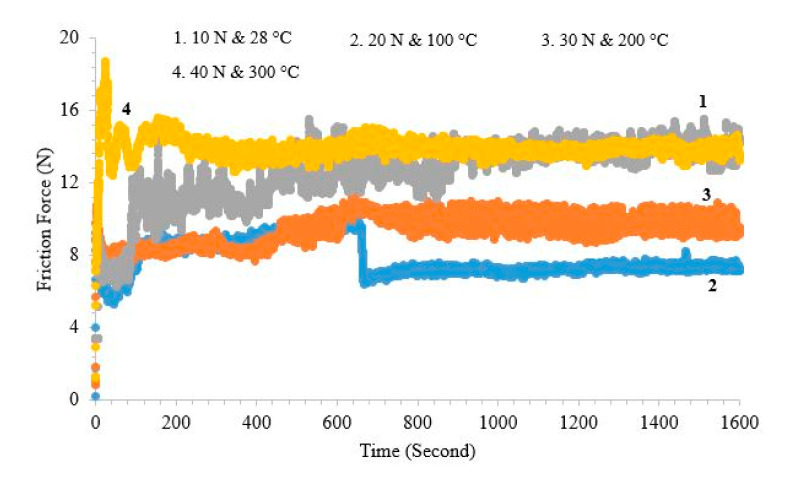
Friction force at a constant speed with increasing load and temperature.

A reciprocating pin-on-disc tribometer was used for frictional force and coefficient of friction (COF) as per the application of study for more appropriate results, while the primary literature used a unidirectional pin-on-disc for the similar application. Friction coefficient values are plotted in Figure 7. At room temperature, i.e., at 25 °C and load 10 N the coefficient of friction is recorded 0.93 at a sliding speed of 0.3 m/s. When temperature and load are increased successively up to 100 °C and 20 N, the value of the friction coefficient that reached a steady-state is 0.41 for a fixed sliding speed (0.3 m/s). Further, the next level increase in temperature and load up to 200 °C and 30 N, the friction coefficient was 0.39. At the maximum temperature, i.e., 300 °C of this study, the friction coefficient was 0.34. The trend of the frictional force is also reported in Figure 8 ranges from 7.81 to 13.94 N with the rise in temperature from 25 °C to 400 °C. One of the interesting patterns can be observed in Figure 7 and Figure 8, a sudden drop in the friction force at 600 s, while at the instant there is a peak formation in the pattern of COF, further stability can be observed. This is due to the fact of some that accumulated mass (wear debris) at the point of contact becomes more stable and results a decrease in friction force, while a disturbance can be seen in the COF pattern due to vibrations of the wear test setup.

It is observed clearly from the above results that the increase in temperature and load is directly affecting the COF. With the increase in temperature that can enhance the heat moving capacity of moving parts, it also causes to increase the molecular distance. The state of change of molecular motion in the view of dynamics is referred to as laxation. Accompanied by the laxation of the CrN coating, the physical properties, thermal properties, and visco-elastic properties would be affected. The coefficient of friction is influenced by the rise of temperature. At the room temperature of 25 °C on the CrN coating and oxide, film was not formed, indicating an interlocking of the metal transfer mechanism, which results in a quite high COF. Whereas at high temperatures (200–300 °C), the transfer film growth and melting of accumulated wear debris become permanent, which is reflected in the low values of friction coefficient from 0.39 to 0.34. Similar trend also reported that the value of COF would be lower at higher temperature due to the adhesion theory. This decrease in COF was also due to the conversion of friction into the heat energy, in addition to providing the required energy for tearing and partial melting of the coating. In addition, the temperature rose due to friction, as reported, reducing the friction coefficient associated with lubricous chromium oxide layer formation [17,18,19].

### 3.4. Wear Mechanism of Coating

**Figure 9:**
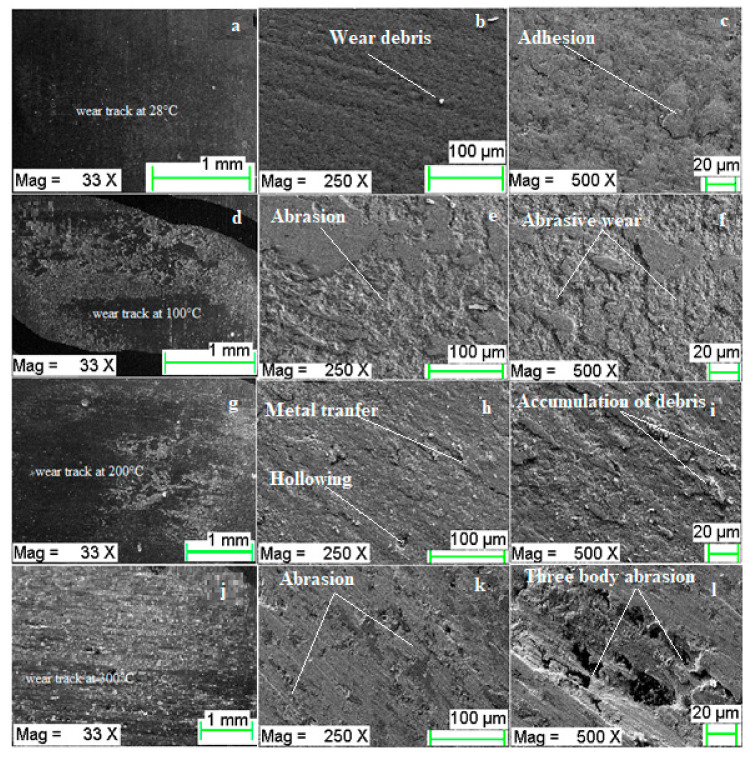
Worn surface morphology of CrN at different test conditions: (**a**–**c**) wear track at 28 °C and 10 N load, (**d**–**f**) wear track at 100 °C and 20 N load, (**g**–**i**) wear track at 200°C and 30 N load, (**j**–**l**) wear track at 300 °C and 40 N load.

Worn surface of the CrN has been investigated by FE-SEM at different magnification to predict the characteristics nature of wear mechanism. Figure 9a shows the worn-out surfaces at room temperature (28 °C), and Figure 9b,c shows the magnified images of wear track. Worn out surface manifest some wear debris with small scratches in the direction of metal transfer. At room temperature, the scratches are almost negligible (Figure 9b) for CrN coating. Some micro-scale cohesive features (Figure 9c) are governed with the potential contribution of an abrasive mechanism of coating at room temperature. This is due to the accumulation of wear debris and smearing of transferred material at the contact occurs where the gap between the CrN coating and counter-body (cylinder liner) surfaces becomes lesser.

While load and temperature are increasing successively up to 20 N and 100 °C, the wear mechanism is manifested with several small grooves, partially at coating surface in Figure 9d–f. Due to the rise in temperature and load, the nitride layers break and accumulation of iron takes place. Irregular hollowing can also be observed due to the combined effect abrasion and adhesion wear mechanism of CrN coating during wear test. While load and temperature both increased up to 30 N and 200 °C, the built-up formation takes place due to the re-deposition of wear debris originated from an eroded layer of CrN and iron from the wear track and counter-body, respectively. The accumulation of wear debris between the CrN and cylinder liner results in three-body abrasion during the wear test. The main wear mechanisms at high temperatures were material-removal-accompanied by oxidative wear. This means that amount of nitrogen transfer present in coating had an effect on tribo-oxidation. This is due to the fact that native oxide layers are self-limiting, most of the oxidations are likely occurred during the mixing of the wear elements because of the higher operating temperatures of the mating parts. It is also evident from the micrographs that degradation of CrN material takes place due accumulation of wear debris results in three body abrasion wear mechanism. Coating at high temperature exhibits lower wear resistance, and accumulation of chromium and iron-oxide at the deep groove was confirmed by FE-SEM observations. Figure 9j shows the wear track at 40 N load and 300 °C, almost maximum removal of Cr and with the decomposition of nitrogen and accumulation of iron took place. The large grooves and erosion of coating are visible in Figure 9l. At 300 °C, the CrN coating also manifests peeling under the action of oxidation takes place at a high temperature and micrograph of the worn track showed furrows and oxides.

The worn surface morphology of oxy-nitride and nitride characterized by the build-upof wear debris, whereas hollowing out was due to erosive action and build-up due to re-deposition reported by the authors [20]. The dominant wear mechanism at atmospheric temperature is abrasive wear accompanied to the possible part of minute cohesive features [21]. There might be a primary chipping point beyond which wear-resistant of the coating drops considerably CrN coatings deposited by PVD under normal operation conditions [22,23,24]. The major wear mechanisms reported at higher temperatures are adhesive wear accompanied with the oxidative wear [25,26]. The similar results also reported about the wear of CrN is abrasive at 300 °C, 400 °C, while at 500 °C abrasive is also accompanied by the light oxidative wear [27].

**Figure 10:**
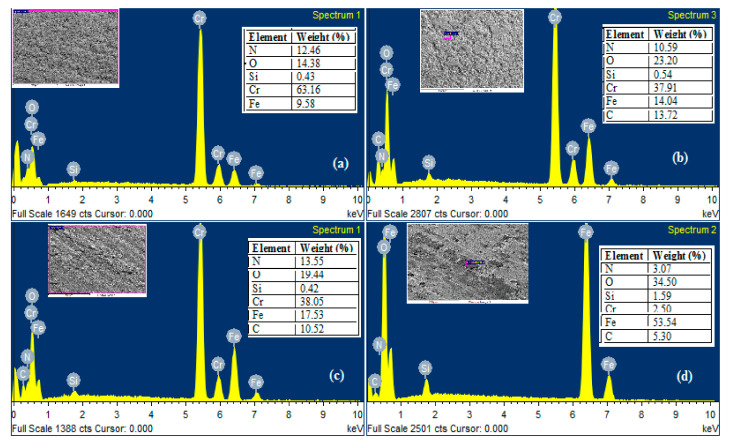
EDS spectra of wear track: (**a**) wear track at 28 °C, (**b**) wear track at 100 °C, (**c**) wear track at 200 °C, (**d**) wear track at 300 °C.

Figure 10 reports the EDS spectra and the weight percentage of the element present in each test from room temperature to 300 °C. Figure 10a shows the scanned area results of CrN wear track at 28 °C in weight %, Cr 63.16, N 12.46, O 14.38, and Fe 9.50. The 9.50% by weight iron transfer from the counter-body at the coating and the wear track was also enriched by the oxygen from 6.42% (before weartest) to 14.38. The Cr and N on the worn track noted 75.62%, indicating that wear was relatively mild at the room-temperature and 10 N load. Figure 10b shows the EDS analysis of the worn surface of coating at 100 °C and 20 N and confirms in weight % Cr37.91, N10.59, C13.72, O23.20, and Fe14.04. The chromium and nitrogen accounted for 48.50% by weight. The Cr and N on the wear track showed the atom poor zone of Cr, indicating that wear was relatively serious. However, the content of nitrogen was slightly changed. The oxygen content increased from 6.42 (before wear test) to 23.20 at 100 °C. The occurrence of the oxidation reaction and the formation of chromium oxide determined due to the presence of a considerable amount of oxygen. EDS scan results and spectra of wear track at 200 °C and 30 N, shown in Figure 10c. The scan results in weight%, 38.05Cr, 13.55N, 19.94O, 10.52C, and 17.53Fe. The nitrogen content had not affected major while the chromium poor zone can be seen on the wear track nearly similar as 100 °C. The oxygen accounted for 19.94% by weight of total concentrations, the oxidation reaction increases, and the presence of hard debris (Figure 10d) is confirmed.

Transfers of iron also take place from counter-body and increase with the increment of temperature in each test. This may increase the furrows formed on the wear track and results in higher wear depth and width. Figure 10d shows the EDS scan results and spectra at 300 °C and 40 N, N3.07, Fe53.54, O36.50, C5.30, and Cr2.50 by weight%. The coating shows a major decrease in the contribution of chromium and nitrogen and rapid increase in iron and oxygen comparatively to 100 °C and 200 °C. The carbon content is continuously decreasing by an increase in temperature during each test.

### 3.5. Wear Mechanism of Counter-Body

**Figure 11:**
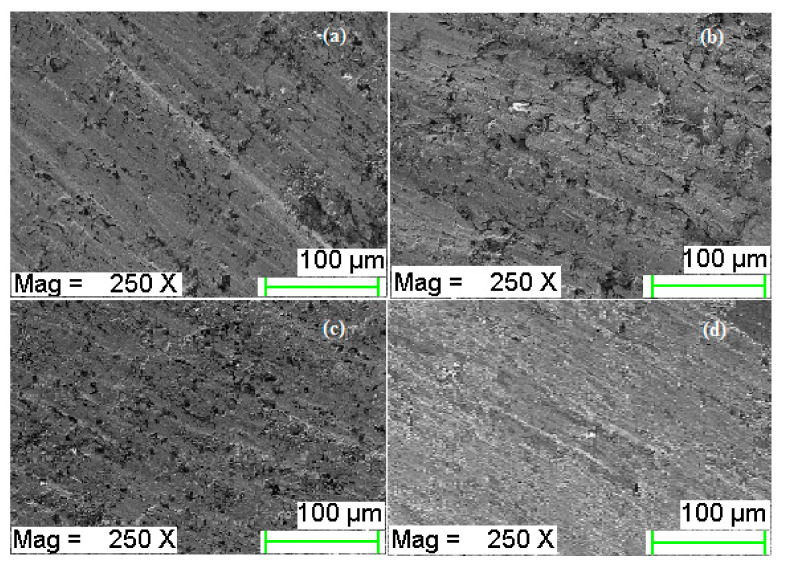
(**a**) Counter-body micrograph after wear test at 28 °C and 10 N, (**b**) counter-body condition after wear test at 100 °C and 20 N, (**c**) counter-body condition after wear test at 200 °C and 30 N, (**d**) counter-body condition after wear test at 300 °C and 40 N.

**Figure 12:**
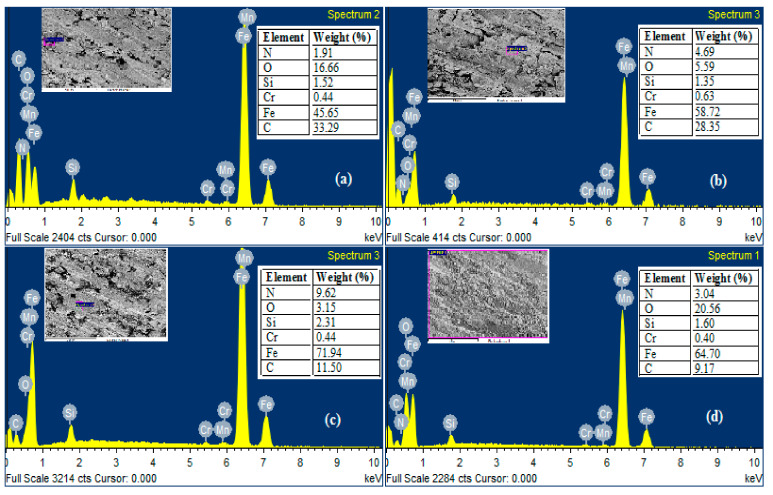
EDS spectra of counter-body: (**a**) wear track of counter-body at 28 °C, (**b**) wear track of counter-body at 100 °C, (**c**) wear track of counter-body at 200 °C, (**d**) wear track of counter-body at 300 °C.

Wear mechanism of the coating is always reliant on the composition and wear nature of counter-body, i.e., made of the cylinder liner in this study. Figure 11a shows the micrograph of counter-body at room temperature (28 °C), Figure 11b–d reports the FE-SEM images of counter-body at 100 °C, 200 °C, and 300 °C. A counter-face after the wear test at room temperature showed some small grooves and transfer of nitrogen and chromium from the coating reported in Figure 12a; the dominating element is iron as it was before wear test, but the contribution of oxygen increased up to 16.66% by weight. The wear track micrograph does not show a major difference after wear test at 200 °C and 300 °C in dry conditions. The cylinder liner top layer relocation can be observed due to the rubbing and plastic deformation at the contact surfaces in the form of lobes. Surfaces conditions confirm that the plastic deformation and ductile shearing at temperature 200 °C and 300 °C, respectively, contain wave-like lobes, while the element composition and spectra are not similar, as shown in Figure 12b,c. It is found that proportion of the carbon 29.82 (before wear test) decreased up to 28.35 (at 100 °C) and 11.50 (at 200 °C), whereas the proportion of iron (Fe) is improved and also added to the minor increment of O_2_, Cr, and Si. The above-mentioned results of EDS confirm that the diffusion of carbon to the coating from the cylinder liner material as a counter-body and that experienced oxidation in addition to the diffusion of chromium from the coating. At 300 °C, the composition of counter-body after the wear test is 9.57% carbon, 20.56% oxygen, and 3.04% nitrogen are present by weight, as shown in Figure 12d.

Similar flakes as counter-body (FE-SEM) have been reported in the literature. The layer looks hard and white at the top that cannot make strength in the wear track at the same time because of the effect of applied normal load making that layer quickly break, thus it is unable to effectively slow down the effect of matrix wear [28,29,30].

## 4. Conclusions

This work presents a study of chromium nitride coating against cylinder liner material at engine operating temperature under reciprocating conditions. Following conclusions can be made:

(1) The chromium nitride coating primarily composed of a small grain and hard phase of CrN, the thickness of 17.93 µm with a contribution of 63.56% chromium and 20.18% nitrogen by weight. Coating shows very good bonding with the substrate, associated with diffusion bonding.
(2) The average value of the friction coefficient of CrN coating against the cylinder liner at 28 °C, 100 °C, 200 °C, and 300 °C is 0.93, 0.41, 0.39, and 0.34, respectively. The average friction coefficient of the tested CrN coating decreased mainly because the smooth hard layer gradually formed on the worn surface of the coating.
(3) The specific wear rate of the coating was 3.15 × 10^−6^ gm/N·m, 2.67 × 10^−6^ gm/N·m, 1.69 × 10^−6^ gm/N·m, and 0.612 × 10^−6^ gm/N·m at 28 °C, 100 °C, 200 °C, and 300 °C, respectively. The formation of chromium-oxide film and re-deposition of hard wear debris plays an important role in the reduction in the wear rate and friction coefficient.
(4) The wear mechanism of CrN coating is mild and at 28 °C and 10 N load, the wear mechanism was mainly adhesive wear, accompanied with three-body abrasion. From 28 °C to 100 °C, the wear mechanism included abrasive wear and oxidation wear. From 200 °C to 300 °C, the wear mechanism of CrN was mainly oxidation wear and abrasive wear.

## Figures and Tables

**Table 1:** Parameters used in tribological testing.

Serial No.	Load (N)	Temperature (°C)	Frequency (Hz)	Stroke Length (mm)	Speed (m/s)	Sliding Distance (m)
Test-1	10	28°C	30	5	0.3	500
Test-2	20	100°C	30	5	0.3	500
Test-3	30	200°C	30	5	0.3	500
Test-4	40	300°C	30	5	0.3	500
